# Annual acknowledgement of manuscript reviewers

**DOI:** 10.1186/1756-0500-6-92

**Published:** 2013-03-27

**Authors:** Emilie Aime

**Affiliations:** 1 BioMed Central, Floor 6, 236 Gray’s Inn Road, London WC1X 8HB, UK

## Abstract

**Contributing reviewers:**

The editors of *BMC Research Notes* would like to thank all of our reviewers who have contributed to the journal in volume 5 (2012).

## 

Fekadu Abebe

Norway

Shahram Abootalebi

Canada

Olivia Achonduh

Cameroon

Bronwen Ackermann

Australia

Marta Ackers

USA

Jeffrey Actor

USA

Ishag Adam

Sudan

Emily Adams

Netherlands

Phil Adams

UK

Lucile L Adams-Campbell

USA

Moses P. Adoga

Nigeria

Syeda Afroze

USA

Ankit Agrawal

USA

Andrés Agudelo-Suárez

Spain

Germán Rubén Aguilar-Gutiérrez

Mexico

Zubair Ahmed

UK

Shahin Akhondzadeh

Iran

Oluwarotimi Akinola

Nigeria

Orhan Aktas

Germany

Tatsuya Akutsu

Japan

Isin Akyar

Turkey

M Firoz Alam

Saudi Arabia

Abebe Alemu

Ethiopia

Helene Alexanderson

Sweden

Waleed Al-Herz

Kuwait

Adnan Al-Hindi

Palestinian Territory

Muhammad Ali

Pakistan

Shawkat Ali

Bangladesh

Gjumrakch Aliev

USA

Jean-Pierre Allain

UK

Irina Alymova

USA

Najith Amarasena

Australia

Patrizia Ambrogini

Italy

Marcel Amills

Spain

Neda Aminzadeh

Iran

F Anagnostopoulos

Greece

Gashaw Andargie

Ethiopia

Jens Peter Andersen

Denmark

Stuart Anderson

UK

K Ang

UK

Giulia Antoniali

Italy

Constantine Antonopoulos

Greece

Halina Antosz

Poland

Atsuko Aoyama

Japan

Katherine Appleton

UK

Ana Aransay

Spain

David Armstrong

USA

Danilo Arnone

UK

Madhumalar Arumugam

India

Samuel Arvidsson

Germany

Adam Asarch

USA

Abraham Aseffa

Ethiopia

Johannes Attems

UK

Matias Attene Ramos

USA

Niyi Awofeso

Australia

Inge Axelsson

Sweden

Duncan Ayers

Malta

Horacio Bach

Canada

Lutz Bachmann

Norway

Steffen Backert

Ireland

Anders Baerheim

Norway

Robert Bailey

USA

Marc Bailly-Bechet

France

Omran Bakoush

Sweden

Alex Balekian

USA

Alexander Ball

UK

Ibrahim Banat

UK

Akash Bang

India

Cecily Banura

Uganda

Ancha Baranova

USA

Rosemary Barber

UK

Ante Barišić

Croatia

Andrea Barison

Italy

Nigel Barker

South Africa

Thierry Baron

France

Roberto Antonio Barrero

Australia

William Barry

USA

Carla Fernanda Barsalobres-Cavallari

Brazil

Rupert Bartsch

Austria

Sebastian Bassi

Argentina

Nahla Bassil

USA

Manuel Battegay

Switzerland

Deepak Batura

UK

Sebastian Bauer

Germany

Bernard Beall

USA

John Beck

USA

Helen Bedford

UK

Mark Behlke

USA

Tim Beissbarth

Germany

Jeffrey Bell

USA

Paolo Bellavite

Italy

M. Eric Benbow

USA

Jason Beneciuk

USA

Abdulbari Bener

Qatar

Mario Bengtson

USA

Teresa Bento

Portugal

Carmen Berasain

Spain

Eva Bergstraesser

Switzerland

James Berkley

Kenya

Fumio Bessho

Japan

Teresa Beynon

UK

Renu Bharadwaj

India

Premananda Bharati

India

Subraya Bhat

India

Eesh Bhatia

India

Jose Ramon Bilbao

Spain

Luigi Bisceglia

Italy

Marcio Sommer Bittencourt

USA

Thomas Bjarnsholt

Denmark

Eva Bjerre Ostenfeld

Denmark

Jonas Bjorkman

Australia

Gunnar Aksel Bjune

Norway

Patrícia Boer

Brazil

Anthony Bolger

Germany

Roumiana Boneva

USA

Viboon Boonsarngsuk

Thailand

Maureen Boost

Hong Kong

Rolf Boot

Netherlands

Signe Borgquist

Sweden

Katherine Borkovich

USA

David Bosanquet

UK

Daniel Bottomly

USA

Laid Boukraa

Algeria

Alida Bouris

USA

Deborah Bowen

USA

Kayvan Bozorgmehr

Germany

Jorgen Bramness

Norway

Claudio Brancolini

Italy

Filipovic Branka

Serbia

Jeff Breckon

UK

Eric Brenner

USA

Romain Briandet

France

Nele Brusselaers

Belgium

Rebecca Bunnell

Kenya

Colleen Burge

USA

Shawn Burgess

USA

William Bush

USA

Larry Bush

USA

Patrick Butaye

Belgium

Andrew Byrnes

USA

Alberto Caban-Martinez

USA

Jean- François Cadranel

France

Sebastien Cagnol

Canada

Zhigangcai Cai

USA

Raffaele Calogero

Italy

Ana G. Camacho

Spain

Antonio Campos

Brazil

Dexter Canoy

UK

Andrea Caporali

UK

Alejandra Caqueo-Urizar

Chile

Gaetano Caramori

Italy

Andres Carrillo

Greece

Anna Cristina Carvalho

Italy

Michele Cassano

Italy

Vincent Cassone

USA

Ufuk Celikkol Akcay

Turkey

Nuno Cerca

Portugal

Miguel Cervero Jimenez

Spain

Alejandro Chade

USA

Wanpen Chaicumpa

Thailand

Neal Chamberlain

USA

Edward Chan

Hong Kong

Partha Chandra

USA

Yuan-Chin Chang

Taiwan

Irini Chatziralli

Greece

Michel Chavance

France

Susana Chavez-Bueno

USA

Norberto Chavez-Tapia

Mexico

Li Chen

USA

Jen-Chih Chen

Taiwan

Guanjie Chen

USA

Marcelo Chen

Taiwan

Hsuanyu Chen

Taiwan

Jinping Chen

China

Jiann-Hwa Chen

Taiwan

Yung-Chang Chen

Taiwan

Junwen Chen

Australia

Patrick Chene

Switzerland

Xuanhong Cheng

USA

Jactty Chew

Australia

Chih-Yi Chien

USA

Chibuike Chigbu

Nigeria

Chandeshwari Chilampalli

USA

Jin Young Choi

USA

Sang-Bong Choi

South Korea

Young Ki Choi

South Korea

Jae Chol Choi

South Korea

Augustine Talumba Choko

Malawi

George Chong

Canada

Siow Ann Chong

Singapore

Ingrid Christiaans-Dingelhoff

Netherlands

Julian Christians

Canada

Dimosthenis Chrysikos

Greece

Chinwe Chukwuka

Nigeria

Ogoamaka Ifeagachukwu Chukwuogo

Nigeria

Yeun-Jun Chung

South Korea

Gaetano Ciancio

USA

Mehmet Ulas Cinar

Germany

M. Gonzalo Claros

Spain

André Clément-Demange

France

Barbara Coco

Italy

Roger Cole

USA

Clare Collins

Australia

Ana Conesa

Spain

Sarah Conklin

USA

Georg Conrads

Germany

Mary Coolbaugh-Murphy

USA

Gertrudes Corção

Brazil

Felipe Cortés Ledesma

Spain

Yvonne Cossart

Australia

Maxime Crochemore

UK

William Crum

UK

Henrique Borges Da Silva

Brazil

Alda Maria Da-Cruz

Brazil

Hannah Dada-Adegbola

Nigeria

Jacques Daele

Belgium

Jesper Dahlgaard

Denmark

Collet Dandara

South Africa

Mylene Dandavino

Canada

Michael David

USA

Evelyn Davila

USA

Boyd Davis

USA

Philip Day

UK

Thierry De Baere

Belgium

Joost De Bruijn

UK

Margaretha Christine De Bruijne

Netherlands

Beatriz De Camargo

Brazil

Simone De Jong

USA

Carolina De Oliveira

Brazil

Satiro De Oliveira

USA

Matty De Wit

Netherlands

Thomas Deleuran

Denmark

Nicolas Demartines

Syria

Marcos Demasi

Brazil

Omer Demir

Turkey

Sharon Demorrow

USA

Jonathan Dennis

Canada

Karthik Devarajan

USA

Ilker Devrim

Turkey

Michael K Deyholos

Canada

Randall Deyoung

USA

Maryam Didehdar Ardebil

India

Francisco Javier Diego-Rasilla

Spain

Constantine Dimitrakakis

Greece

Ivanka Dimova

Bulgaria

Petros Dinas

Greece

Mamuka Djibuti

Georgia

Riitta Dlodlo

Zimbabwe

Alexander Dobrovic

Australia

Fernanda Domingues

Portugal

M. Angeles Dominguez

Spain

Guo Dongsheng

Sweden

Helen Donoghue

UK

Sergey Dorozhkin

Russian Federation

Horng-Yunn Dou

Taiwan

Bedis Dridi

Canada

Oana Dumitrescu

France

Tim Dumonceaux

Canada

Richard Duncan

New Zealand

Caroline Durif

Norway

Chandradhar Dwivedi

USA

Anders Bondo Dydensborg

Canada

Jose Echevarria

Spain

Assimoula Economopoulou

Sweden

Inger Edfors

Sweden

Konstantinos Efthimiou

Greece

Anna Mia Ekström

Sweden

Gaber El-Baradei

Egypt

Abdel-Hady El-Gilany

Egypt

Avishay Elis

Israel

Niklas Elmqvist

USA

Pinar Erbay Dündar

Turkey

Jenni Ervasti

Finland

Ricardo Escalante

Spain

Juan Carlos Espinosa

Spain

Ian Evans

UK

Chris Exley

UK

Nancy Fahrenwald

USA

Kuangnan Fang

China

Fan Fang

China

Christian Fankhauser

Switzerland

Mehdi Farhanghi

Iran

Reza Farhud

Iran

Abid Farooqi

Pakistan

Marie-Anne Felix

France

Fran Feltovich

USA

Quan Zhou Feng

China

Jorge Fernandes

Norway

Paulo Ferreira

Brazil

Gregg Fields

USA

Taciane Finatto

Brazil

Fiona Finlay

UK

Vincent Fischetti

USA

Frank Flachskampf

Sweden

John Fletcher

Australia

Virginia Fonner

USA

Paolo Fontana

Italy

Monica Forni

Italy

Simon Foster

UK

Neil Fowler

UK

Brian Fox

USA

Bart Frank

USA

Thomas Charles Freeman

UK

Sebastien Fribourg

France

Jan C. Frich

Norway

Steve Frost

Australia

Xueyan Fu

USA

Shu-Ling Fu

Taiwan

King-Wa Fu

Hong Kong

John Fuerst

Australia

Jens Gaab

Switzerland

Eduardo Gaio

Brazil

Efie Galiatsou

Greece

Moses Galukande

Uganda

Rebecca Ganann

Canada

Vijay Ganji

USA

Fan Gao

USA

Enrico Garattini

Italy

Stephen Garba

Nigeria

Dana Garcia

USA

Pilar García

Spain

Jose F Garcia-Mazcorro

Mexico

Ana L. Garcia-Perez

Spain

Jonathan Gardner

New Zealand

Junkal Garmendia

Spain

Michael Garrett

USA

Emilio Garrido

USA

Gail Gasparich

USA

Laurent Gatto

UK

Daniel Gaudet

Canada

Raif Geha

USA

Karen Gerlach

USA

Joseph Geskey

USA

Constance Gewa

USA

Arash Ghanbarian

Iran

Maibritt Giacobini

Sweden

Christoforos Giannaki

Cyprus

Georgios Giannakoulas

Greece

Yan Gilbert

Canada

Janet Gilsdorf

USA

Cinzia Giuli

Italy

Tor Gjøen

Norway

Shimon Glick

Israel

Greg Gloor

Canada

David Gomez-Cabrero

Spain

Marilda Goncalves

Brazil

Javier Gonzalez

Spain

Patricia Gonzalez-Moreno

Mexico

Guduru Gopal Rao

UK

Dimitrios Goulis

Greece

Evanthia Gouveri

Greece

Elmar Graessel

Germany

Claudia Gragnoli

USA

Stephen Graves

Australia

Stuart Gray

UK

Celia Greenwood

Canada

Philip Gregory

USA

Daniel Grenier

Canada

Margherita Grotzkyj Giorgi

UK

Andrzej Grzybowski

Poland

Biancamaria Guarnieri

Italy

Simon Gubbins

UK

Tore Gude

Norway

Jean Louis Guenet

France

Mustafa Gul

Turkey

Dilek Guldal

Turkey

Teresa Gunn

USA

Revathi Gunturu

Kenya

Christine Guptill

Canada

Vsevolod Gurevich

USA

Lyle Gurrin

Australia

Padmalal Gurugama

UK

Gabriel Gutkind

Argentina

Francesco Guzzetta

Italy

Patsy Haccou

Netherlands

Ray Hachem

USA

Georgios Hadjigeorgiou

Greece

Randi Hagerman

USA

Katsuro Hagiwara

Japan

Martin Hahn

Austria

Jemal Haidar

Ethiopia

Batool Haider

USA

Mahmoud Halablab

Lebanon

Rahman Hallaj

Iran

Yuepeng Han

China

Scott Handley

USA

Wiebke Hansen

Germany

Brendon Hanson

Singapore

Thomas James Hardcastle

UK

Jeffrey Hargrove

USA

Guy Harling

USA

Wendy Harwood

UK

Anna Haukioja

Finland

Robert Hausinger

USA

Stephen Hawes

USA

Dave Hayes

Canada

Kun He

USA

Ji He

USA

Zhiwei He

China

Fei He

China

Jeremy Hebden

UK

Ali Morad Heidari Gorji

Iran

Marleen Hendriks

Netherlands

Helen Heneghan

Ireland

Birgit Henrich

Germany

Christiane Herden

Germany

Henning Hermjakob

UK

Paul Hernandez

USA

Montserrat Hervella

Spain

Christoph Hiemke

Germany

Roger Hilfiker

Switzerland

Nicole Hoefsmit

Netherlands

Stefan Hohmann

Sweden

Philip Holmes

USA

Carol Holm-Hansen

Norway

Harriet Hopf

USA

Juan Pablo Horcajada

Spain

Mads Hornum

Denmark

Matthew Hotopf

UK

Josef Houštěk

Czech Republic

Chen-Hsi Hsieh

Taiwan

Zhibin Hu

China

Chunfa Huang

USA

Joel Huberman

USA

Christer Hublin

Finland

Eva Hummers-Pradier

Germany

Ling-Hong Hung

USA

Nora Hunter

UK

Johan Hviid Andersen

Denmark

Kenji Ichiyanagi

Japan

Kayode Ijadunola

Nigeria

Anthony Ikefuna

Nigeria

Fumiaki Ikeno

USA

Toshihiko Imamura

Japan

Shinsaku Imashuku

Japan

Mercedes Iñarrairaegui

Spain

Seema Irfan

Pakistan

Karin E Isaksson Ro

Norway

Takuma Ishizaki

Japan

Md. Taohidul Islam

Bangladesh

Takao Iwawaki

Japan

Sandra Iwuala

Nigeria

Kauser Jabeen

Pakistan

Kathryn H. Jacobsen

USA

Sandra Jacobson

USA

Johannes Jaeger

UK

Mukesh Jain

India

Bawo James

Nigeria

Shazia Jamshed

Malaysia

Jan Janecka

USA

Ziad Jaradat

Jordan

Ashwath Jayagopal

USA

Shireen Jejeebhoy

India

Zhu-Qiu Jin

USA

Sigrun Johannesdottir

Denmark

David Johnson

Australia

Leigh Johnson

South Africa

Charles Johnson

USA

Moses Joloba

Uganda

Andrew Jones

UK

Graham Jones

Australia

Colleen Jonsson

USA

Francisco Jover

Spain

Carlos Juan Nicolau

Spain

Petro Julkunen

Finland

Klaus Jung

Germany

Ki-Hong Jung

South Korea

Christopher Justinich

Canada

Helle Juul-Madsen

Denmark

Koji Kadota

Japan

Michael Kahn

USA

Hironaga Kakoi

Japan

Anna Kaltsouda

Greece

Umesh Kamat

India

Maged N. Kamel Boulos

UK

Biao Kan

China

Satoko Kanematsu

Japan

Sung Ung Kang

USA

Eva Johanna Kantelhardt

Germany

Neena Kanwar

USA

Rowland Raymond Kao

UK

Evangelos Karademas

Greece

Jens Kastrup

Denmark

Harparkash Kaur

UK

Radhey Shyam Kaushik

USA

Sarah Kehoe

UK

Marcus Kehrli

USA

Michael Kertesz

Australia

Peter Kevern

UK

Amir Khan

Ireland

Asad Khan

Australia

Muhammad Asim Khan Rehmani

Pakistan

Yasmin Khatib

UK

Fatemeh Khoshkhou

Canada

Eun-Kyu Kim

South Korea

Tae Won Kim

South Korea

Andrew Kingsnorth

UK

Akira Kinjo

Japan

Andrew Kinninmonth

UK

Shintaro Kinugawa

Japan

John Kinuthia

Kenya

Ioannis Kioumis

Greece

Maggie Kirkman

Australia

Seiichiro Kiyota

Japan

Christian Klatt

Sweden

Jeffrey Klausner

USA

David Klemperer

Germany

Carolyn Klinge

USA

Marian Klinger

Poland

Anita Kloss-Brandstaetter

Austria

Birthe Loa Knizek

Norway

Steen Knudsen

Denmark

Vibeke Kildegaard Knudsen

Denmark

Toshiki Kobayashi

USA

Thomas Köcher

Austria

Joseph Koke

USA

Gerasimos Kolaitis

Greece

John Kolega

USA

Tomas Koller

Slovakia

Altaf Kondkar

Saudi Arabia

Masahiro Kono

USA

Anastassia Kossioni

Greece

Sundar Rao Koyyalamudi

Australia

Tadahiko Kozu

Japan

Konstantinos Krampis

USA

D Kreil

Austria

Hans Kreipe

Germany

J Krishnan

UK

Gv Krishnaveni

India

Ajit Kulkarni

USA

Ambuj Kumar

India

Florian Kurth

USA

Juan Kusanovic

USA

Eiji Kutoh

Japan

Lance Kwok

Australia

Kwang-Il Kwon

South Korea

Gilles Labesse

France

Salil Lachke

USA

Suzanne Lagerveld

Netherlands

Chien-Tsai Lai

Canada

Dennis Landin

USA

Xiang Qian Lao

Hong Kong

Ismael Lares-Asseff

Mexico

Bernard Larouze

France

David Larsen

USA

Suzie Lavoie

Australia

Ck Law

Hong Kong

Lucinda Lawson

USA

Rosario Le Moli

Italy

Amanda Leach

Belgium

Amanda Leach

Australia

Yun-Shien Lee

Taiwan

Yin-Won Lee

South Korea

Eugene Leibovitz

Israel

Forian Leitner

Spain

Giorgio Lenaz

Italy

A. Martin Lerner

USA

Olufunmilayo Lesi

Nigeria

Roni Levy

Israel

Dinorah Leyva

USA

Xiaohong Li

USA

Zhijun Li

USA

Zheng Li

USA

Jingyi Li

USA

Zhen Li

China

Mengnai Li

USA

Kai Li

China

Anatoly Lichtenstein

Russian Federation

Charles Lidz

USA

Ruta Liesiene

Lithuania

Mark Lin

USA

Hung-Du Lin

Taiwan

Wen-Yuan Lin

Taiwan

Jan Erik Lindberg

Sweden

Bernt Lindtjorn

Norway

Georg Linke

Germany

Christos Lionis

Greece

Břetislav Lipový

Czech Republic

Lara Lipton

Australia

Zhi-Ping Liu

China

Mei Liu

USA

Chen-Chung Liu

Taiwan

Chien-Tsai Liu

Taiwan

Jin Liu

USA

Fu-Tong Liu

USA

Shu-Qun Liu

China

Chandrani Liyanage

Sri Lanka

Augusto Llosa

France

Luca Longo

Italy

Amy Lossie

USA

Donald Love

New Zealand

Andrew Lover

Singapore

Qiang Lu

China

Aiping Lu

China

Beibei Lu

USA

David Lumenta

Austria

Cheng Luo

China

Mark Lutman

UK

Shuangge Ma

USA

Deborah Mackay

UK

Colin Rae Mackenzie

Germany

Michael Mackert

USA

Allison Madigan

USA

Irene Madrigal

Spain

Matthew Magee

USA

Franco Maggiolo

Italy

Konstantina Magklara

Greece

Duhita Mahatmya

USA

Sajjad Mahmood

UK

Chistophe Maïano

Canada

Christoph Maier

USA

Tomas Maira-Litran

USA

Reza Majdzadeh

Iran

Paromita Majumder

UK

Antti Mäkitie

Finland

Dimitrios Makridis

Greece

Thibaut Malausa

France

Charles Malemud

USA

Gabriella Malfatto

Italy

Sumit Malhotra

India

Nancy Malla

India

Massimo Mallardo

Italy

Sarmila Mallik

India

Romina Mancinelli

Italy

Martin Mangino

USA

Alexandre Manirakiza

Central African Republic

Johannes Mann

Germany

Gwen Manning

Ireland

Magnus Manske

UK

Maria Cristina Marazzi

Italy

Harvey Marcovitch

UK

Marketa Mareckova

Czech Republic

Mauro Mari

Italy

Romain Marignier

France

Jose Marin

Spain

Saša Markovič

Slovenia

Guillermo Andrés Maroniche

Argentina

Pedro Marques-Vidal

Switzerland

Lennart Martens

Belgium

Javier Martin

Spain

Cristina Martínez

Spain

Cynthia Masison

USA

Shogo Matoba

Japan

Frauke Mattner

Germany

Igor Matushansky

USA

Olga Matveeva

USA

Claus Mayer

UK

Leonard Mboera

Tanzania

Francois-Xavier Mbopi-Keou

Cameroon

Zakaria Mbwambo

Tanzania

Steve Mcanulty

USA

Mary Mccaffrey

Ireland

Lesley Mcgee

USA

Andrew Mckechanie

UK

P G Mckenna

UK

Kwame Julius Mckenzie

Canada

Bradley Mcpherson

Hong Kong

Sarika Mehra

India

André Meichtry

Switzerland

Markus Meissner

Germany

Liv Mellesdal

Norway

Maria Cassia Mendes-Correa

Brazil

Fan Meng

USA

Mark Mescher

USA

Jane Messina

USA

Francois Meurens

Canada

Gabriele Meyer

Germany

Eli Meyer

USA

Guerraz Michel

France

Alan Mileham

USA

Andrea Minervini

Italy

Jessica Minion

Canada

Martine Miquel

France

Jose Maria Miranda-Sayago

Spain

Nagendra Mishra

USA

Dimitris Missirlis

Germany

Anna Paola Mitterhofer

Italy

Ikuo Miura

Japan

Makiko Mizuno

Japan

Beyene Moges

Ethiopia

Yashar Moharamzad

Iran

Shiueh Lian Mok

Malaysia

Peter Molan

New Zealand

Ali Montazeri

Iran

Beronda Montgomery

USA

Jason Moore

USA

Graham Moore

UK

Marcio De Carvalho Moretzsohn

Brazil

Donald Morisky

USA

Steffen Moritz

Germany

Brian Morris

Australia

Joan Morris

UK

Giulia Morsica

Italy

Justin Mott

USA

Ahmed Moussa

Morocco

Raphael Mrode

UK

Godfrey Mubyazi

Tanzania

Christoph Muhtz

Germany

Malay Mukherjee

India

Marie-Eve Muller

Switzerland

Borna Müller

Switzerland

Andargachew Mulu

Ethiopia

Francina Munell

Spain

Maria Munne

Argentina

Shona Murphy

UK

Kennedy Mwambete

Tanzania

Susan Nagel

USA

Gera E Nagelhout

Netherlands

H R Nagendra

India

Vinod Nair

India

Sigve Nakken

Norway

Shigetou Namba

Japan

Tsutomu Namikawa

Japan

Christine Nardini

China

Saminathan Nathan

Singapore

Gerardo Nava

USA

Prasunpriya Nayak

India

Juliet Ndibazza

Uganda

Nick Neave

UK

Saharnaz Nedjat

Iran

Giesje Nefs

Netherlands

Angelo Nespoli

Italy

Sudan Neupane

Norway

Mariana Neves

Germany

Thomas Newton

USA

Cecilia Ng

Australia

Hoa Nguyen B

Vietnam

Conrad Nieduszynski

UK

Liene Nikitina - Zake

Latvia

Francois Niyonsaba

Japan

Polly Noel

USA

Abdollah Noorbakhsh

Iran

Tsering Norboo

India

Paolo Norio

USA

Anna Nowak

Australia

Peter Nthumba

Kenya

Diana Nurutdinova

USA

Fred Nuwaha

Uganda

Eric Obikeze

Nigeria

Chinwe Ogbonna

Nigeria

Graham Ogg

UK

Justin O'Grady

UK

Steffen Ohlmeier

Finland

Colm O'Huigin

USA

Denise O'Keefe

USA

Uchenna Okolie

Nigeria

Ijeoma Okoronkwo

Nigeria

Patricia Okubara

USA

Shujiro Okuda

Japan

Timothy O'Leary

USA

Francesco Oliva

Italy

Jose Luis Oliveira

Portugal

Rúbia Oliveira

Brazil

Brian Olshansky

USA

Pal A. Olsvik

Norway

Chika Onwasigwe

Nigeria

Stephen Onwere

Nigeria

Obinna Onwujekwe

Nigeria

Cajetan Onyedum

Nigeria

Erman Or

Turkey

Hideo Orimo

Japan

Brian Ort

USA

Alex Ortega-Loayza

USA

Elizabeth Orton

UK

Emmanuel Osei Tutu

Ghana

Hibah Osman

Lebanon

Pere P. Simarro

Switzerland

Jorma Paavonen

Finland

Roderic Page

UK

Ewa Paleolog

UK

Ståle Pallesen

Norway

Jose Prisco Palma-Nicolas

Mexico

Archana Pan

India

Tiziana Pandolfini

Italy

Hsing-Kuo Pao

Taiwan

Nikolaos Papanas

Greece

Evridiki Papastavrou

Cyprus

Athanasia Papazafiropoulou

Greece

Dimitrios Papazoglou

Greece

Emmanouil Paraskakis

Greece

Taesung Park

South Korea

Leslie Parker

USA

Cynthia Parr

USA

Geraldo Passos

Brazil

Viraj Patel

USA

Douglas Paton

Australia

Bikash Pattnaik

USA

Gillian Paul

Ireland

Friedemann Paul

Germany

Edit Paulik

Hungary

C. David Pauza

USA

Nick Pavlakis

Australia

Jorge Pedrosa

Portugal

Abraham Peedicayil

India

Sarah Pendergrass

USA

Maria Olivia Pereira

Portugal

Antonio Pereira-Vega

Spain

Kenneth Peterson

USA

Clive J Petry

UK

Kamija Phiri

Malawi

Vorapong Phupong

Thailand

Rosanna Piccirillo

Italy

Sarina Pignato

Italy

John W Pinney

UK

Athanasios Pipilis

Greece

Fraser Pirie

South Africa

Andrzej Plawski

Poland

Ioannis Pneumatikos

Greece

Kathryn Pollak

USA

Patsie Polly

Australia

Palmiro Poltronieri

Italy

Monica Ponder

USA

Georg Pongratz

Germany

Sreenivasan Ponnambalam

UK

Ana Clara Pontaroli

Argentina

Belen Ponte

Switzerland

Mihai Pop

USA

Shinu Pottathil

India

Kalpana Poudel-Tandukar

Japan

Andrew Prendergast

UK

John Prescott

Canada

Luiz Prestes-Carneiro

Brazil

David Preston

USA

Are Hugo Pripp

Norway

Andreas Prokesch

Austria

Jerry Punch

USA

Seema Puri

India

László Puskás

Hungary

Denis Puthier

France

Katarina Putnik

Netherlands

Liu Qiu

China

Matthew Quinn

USA

Giuseppe Quintaliani

Italy

Lars Radke

Germany

Markus Ralser

UK

Anurag Ramavat

India

Ramon Rami-Porta

Spain

Marizen Ramirez

USA

Jose Manuel Ramos

Spain

Aishwarya Rao

India

Raghavendra Rao

India

Mahesh Rao

India

Armin Rashidi

USA

Vagner Raso

Brazil

Jeremy Rassen

USA

Dietrich Rebholz-Schuhmann

Switzerland

Terri Rebmann

USA

Kevin Regner

USA

Christian Reidys

Denmark

Ulf Reineke

Germany

Todd Reinhart

USA

Ricardo Reis

Brazil

Adam Reitzel

USA

Silje Endresen Reme

USA

Xiaofeng Ren

China

Heinrich Resch

Austria

Klaus Reuter

Germany

Jose A. Riancho

Spain

Barbara Riddell

UK

Igino Rigato

Italy

Grigori Rijakov

UK

Eleanor Riley

UK

Monica Rittler

Argentina

Amanda Roberts

UK

John Robinson

Switzerland

Matilde Rodriguez-Cerrillo

Spain

Carlos M. Rodriguez-Ortigosa

Spain

Claire Rogel-Gaillard

France

Wesley Rohrer

Saudi Arabia

Yamila Romer

Argentina

Thomas Romig

Germany

James Roney

USA

Anna Rose

UK

Peter Rosenbaum

Canada

François Rousseau

France

David Rowe

UK

Praveen Roy

USA

Ludek Roznovsky

Czech Republic

Dan Ruderman

USA

Joy Rudland

New Zealand

Enrique Ruiz De Gopegui

Spain

Vivian M. Rumjanek

Brazil

Charumathi Sabanayagam

Singapore

Sreedharan Nair Sabarinath

USA

Ulrich Sack

Germany

Elianna Saidenberg

Canada

Hector Saka

USA

Maria Samsonova

Russian Federation

Susan Sangha

USA

Ann Sangthong

Thailand

Virender Sangwan

India

Anna Sannino

Italy

Francisco Santos-O'Connor

Spain

Silvia Sardi

Brazil

Indra Neil Sarkar

USA

Sivatej Sarva

USA

Helga Sauerwein

Germany

Christos Savopoulos

Greece

Nongyao Sawangjaroen

Thailand

Aldo Scarpa

Italy

Wolfgang Schaden

Austria

Thomas Scheewe

Netherlands

Marco Schilham

Netherlands

Christopher Schlett

Germany

Gerd Schmalisch

Germany

Sebastian Schmeier

New Zealand

Morten Schmidt

Denmark

Georg Schmolzer

Canada

Uwe Scholz

Germany

Katharina Schönberger

Germany

Lilia Blima Schraiber

Brazil

Lode Schuerman

Belgium

Markus Schwarzlander

Germany

Herbert Schweizer

USA

Jason Scibek

USA

Teresa Seefeldt

USA

Terence Seemungal

Trinidad and Tobago

Leila Sekhavat

Iran

Neha Sekhri

UK

Samy Selim

Egypt

Juliet Shaffer

USA

Muamar Shaheen

Palestinian Territory

Md. Shahidullah

Bangladesh

Isdore Chola Shamputa

Canada

Surender K Sharma

India

Pawan Sharma

India

Dawn Sheppard

Canada

Li Shi

China

Yuling Shi

China

Masahiko Shibata

Japan

Takahito Shikano

Finland

Sung Jae Shin

South Korea

Ho Sik Shin

South Korea

Kenta Shirasawa

Japan

Adam Shlien

UK

Rong Shu

China

Ulrich Sigwart

Switzerland

Diego Silva

Brazil

Marcia Silver

USA

Kang Sim

Singapore

Alan Simmons

USA

Dimitrios Sinapis

Greece

Subrata Sinha

India

Sujatha Sistla

India

Petros Skapinakis

Greece

Suzanne Skevington

UK

Frans Sluyter

UK

Ilias Smilios

Greece

Agnes Jacoline Smink

Netherlands

Duncan R. Smith

Thailand

Shubulade Smith

UK

Philip Smith

USA

Lee Smith

UK

Lucy Smith Paintain

UK

Kathy Smith-Dijulio

USA

Raquel Soares

Portugal

Mette Søgaard

Denmark

Miguel A Sogorb

Spain

Aliyah Sohani

USA

Jean-Karl Soler

Malta

Holger Sondermann

USA

You-Qiang Song

Hong Kong

Silvia Viviana Soriano

Argentina

Manuel Soto

Spain

Paul Span

Netherlands

Jonathan Spector

USA

Hélder Spínola

Portugal

Sivashanmugam Dhandapani

India

Joshua Ssebunnya

Uganda

Richard Stabler

UK

Gerhard Steger

Germany

Olof Stephansson

Sweden

William Stone

USA

Rita Strack

USA

Jana Strahler

Germany

Phyllis Strauss

USA

Carmen Stromberger

Germany

Carmelo Lucio Sturiale

Italy

Zhen Su

China

Wei-Juin Su

Taiwan

Cassidy Sugimoto

USA

Athula Sumathipala

Sri Lanka

Gerald Sume

Cameroon

Anne Sumner

USA

Martha Sund-Levander

Sweden

Fung-Chang Sung

Taiwan

Stephane Supiot

France

Charles Swanton

UK

Peter Sykacek

Austria

Ghazi Tadmouri

United Arab Emirates

Akihiro Takemura

Japan

Reiri Takeuchi

Japan

Mohammad Talaei

Iran

Masanori Tamaoki

Japan

Sze Yen Tan

USA

Sudhakar Johnson Tangirala

India

Flora Tassone

USA

Mark Taubert

UK

Guan Tay

Australia

Bineyam Taye

Ethiopia

Peter Taylor

UK

Shirley Telles

India

Bas Teusink

Netherlands

Gerhard Thallinger

Austria

Anastasia Thanopoulou

Greece

Henry Thode

USA

Torsten Thomas

Australia

Shaji Thomas

India

Patricia Thompson

USA

Yin-Jing Tien

Taiwan

Stephenie Tiew

UK

Suresh Tikoo

Canada

Frank-Peter Tillmann

Germany

Claudio Tiribelli

Italy

Nicola Tolliday

USA

Zhaoguo Tong

China

Stefano Toppo

Italy

Juan Maria Torres

Spain

Fernando Torres-Perez

Chile

Ellen Toth

Canada

Tsuen-Chiuan Tsai

Taiwan

Po-Nien Tsao

Taiwan

Boldtsetseg Tserenpuntsag

USA

Alexandra Tsigginou

Greece

Budd Tucker

USA

James K Tumwine

Uganda

Shigeharu Uchiyama

Japan

Nkolika Uguru

Nigeria

Anirudh Ullal

USA

Dominic Upton

UK

Iker Uriarte

Spain

Raquel Urtasun

Spain

Olalekan Uthman

Niger

Sergio Vaca

Mexico

Tushar Vachharajani

USA

Edi Vaisbuch

USA

Yuvaraj Vaithilingam

India

Angelica Valente

Italy

Pieter Van Den Abbeele

Belgium

Adriaan Van Den Brule

Netherlands

Ron Van Den Bussche

USA

Isaäc Van Der Waal

Netherlands

Martie Van Der Walt

South Africa

J.B. (Hans) Van Goudoever

Netherlands

Thomas Van Gulik

Netherlands

Frank Van Leth

Netherlands

Marc Van Regenmortel

France

Arnoud Van Vliet

Netherlands

Arnoud Van Vliet

UK

Timothy Vanwagoner

USA

John Varlotto

USA

Gerardo Vasta

USA

Rossitza Vatcheva-Dobrevska

Bulgaria

James Vaughn

USA

Alison Venn

Australia

Marco Ventura

Italy

Marcel Verheij

Netherlands

Sergio Verjovski-Almeida

Brazil

Indu Verma

India

Silvan Vesenbeckh

USA

Aristidis Veskoukis

Greece

Rajeev Vibhakar

USA

Manuel Vilanova

Portugal

Humberto Villacorta

Brazil

Livia Villar

Brazil

Martin Vingron

Germany

Todd Vision

USA

Mohan Viswanathan

India

Libor Vitek

Czech Republic

Clementine Vitte

France

Pirashanthie Vivekananda-Schmidt

UK

Juan Antonio Vizcaino

UK

Victoriya Volkova

USA

Olaf Von Dem Knesebeck

Germany

Brigitte Von Rechenberg

Switzerland

Wanwipa Vongsangnak

China

Jana Vrbikova

Czech Republic

Raja Vukanti

India

Mihir Wagh

USA

Glenn Walker

Australia

Deeann Wallis

USA

Michael Walther

USA

Jan Walther-Rasmussen

Denmark

Richard Wamai

USA

Tzu-Hao Wang

Taiwan

Chao-Hung Wang

Taiwan

Meng-Ting Wang

Taiwan

Xiyin Wang

USA

Kai Wang

USA

Miriam Warnier

Netherlands

Rory Watt

Hong Kong

Daniel Weber

Germany

Claus Wedekind

Switzerland

Scott Weese

Canada

Zhi Wei

USA

Shannon Weigum

USA

Steffen Weikert

Germany

Jonathan Weiss

Australia

Henrik Westh

Denmark

Heather Wharrad

UK

Stephen White

USA

Erin White

USA

Richard White

USA

Andrew Christopher Whitelaw

South Africa

Melissa Whiteman

USA

Brandy Wicklow

Canada

Shelley Wilkinson

Australia

Jonathan Wilksch

Australia

Charles Williams

USA

Colin Willoughby

UK

Anna Wirtz

USA

Kathleen Wolin

USA

Kenneth Wong

Hong Kong

William Worodria

Uganda

Craige Wrenn

USA

Rex A Wright

USA

Qi Wu

China

Chuntao Wu

USA

Wenzhong Xiao

USA

Luigi Xodo

Italy

Peisheng Xu

USA

Chandra Yallampalli

USA

Masahito Yamada

Japan

Michel Yamagishi

Brazil

Masayuki Yamamoto

Japan

Fengting Yan

USA

Hsin-Chou Yang

Taiwan

Fuquan Yang

China

Shuhua Yang

China

R Yasodha

India

Aymen Yassin

Egypt

Aymen Yassin

USA

Dorothy Yeboah-Manu

Ghana

Kader Yildiz

Turkey

Hsiang Yin

USA

Gui-Shuang Ying

USA

Kyoung-Jin Yoon

USA

Janet York

USA

Jack Youngren

USA

Mirghani Yousif

Sudan

Joshua Yuan

USA

Chao Zhang

USA

Qingzhao Zhang

China

Jitao David Zhang

Switzerland

Qihui Zhu

USA

Ekhard Ziegler

USA

Ray Zielinski

USA

Flavio Zolessi

Uruguay

Martin Zoremba

Germany

Nikos Zourbanos

Greece

Peter Zuber

USA

Mikhajlo K. Zubko

UK

Inge Zuhorn

Netherlands

Alimuddin Zumla

UK

